# Association between smoking habits and dental care utilization and cost using administrative claims database and specific medical check-up data

**DOI:** 10.1186/s12903-022-02397-7

**Published:** 2022-09-02

**Authors:** Kahori Kawamura, Takashi Doi, Keita Kano, Masanori Matsui, Yuka Hattori, Fukutaro Onishi, Hirotsuka Fukata, Tatsuro Miyake

**Affiliations:** 1 Department of Preventive and Community Dentistry, Osaka Dental University, 8-1 Kuzuhahanazono-cho, Hirakata, Osaka 573-1121 Japan; 2 Graduate School of Dentistry (Department of Preventive and Community Dentistry), Osaka Dental University, Osaka, Japan; 3 Japan Health Insurance Association Osaka Branch, Osaka, Japan; 4 Osaka Dental Association, Osaka, Japan

**Keywords:** Smoking habits, Dental care utilization, Dental cost, Administrative claims database

## Abstract

**Background:**

This study aims to evaluate the association between smoking habits and dental care utilization and cost in individuals registered with the Japan Health Insurance Association, Osaka branch.

**Methods:**

We used the administrative claims database and specific medical check-up data and included 226,359 participants, who visited dental institutions, underwent dental examinations, and underwent specific medical checkups, with smoking data from April 2016 to March 2017. We calculated propensity scores with age, gender, exercise, eating habits, alcohol intake, and sleep. We also compared dental care utilization with the total cost of each procedure.

**Results:**

According to propensity score matching, 62,692 participants were selected for each group. Compared to non-smokers, smokers were younger, and a higher proportion were men. Smokers tended to skip breakfast, have dinner just before bed, and drink alcohol. After adjusting for potential confounding factors with propensity score matching, the mean annual dental cost among smokers was significantly higher than non-smokers. The prevalence of pulpitis, missing teeth, and apical periodontitis were higher among smokers than non-smokers, while inlay detachment, caries, and dentine hypersensitivity were higher among non-smokers.

**Conclusion:**

This study suggests that smokers have higher dental cost consisted of progressive dental caries, missing teeth, and uncontrolled acute inflammation that necessitated the use of medications. It is suggested that smokers tend to visit the dentist after their symptoms become severe.

## Introduction

According to the National Health and Nutrition Survey in 2017, the overall prevalence of smoking in Japan is 17.8% (29.0% among men and 8.1% among women)^1^. Tobacco smoking is still one of the leading causes of mortality and morbidity worldwide^2^. Smoking is not only the cause of lung cancer but also a risk factor for various dental diseases such as periodontal disease, oral cancer, caries, and tooth loss, as indicated by several epidemiologic studies^3–6^.

Drilea et al. reported that smokers were less likely to visit dental clinics than non-smokers, when the data were adjusted for age, sex, race/ethnicity, poverty level, dental insurance, and dentate status^7^. As a result, they are more susceptible to oral problems such as dentine hypersensitivity, toothache, and orofacial pain^8^. Another study showed that smokers were more likely to perceive that they had poorer oral health and tended to undergo dental checkups only when symptomatic^9^. Moreover, lower use of dental service among smokers increased the risk of several oral diseases including oral cancer^10^. The use of dental services after the appearance of symptoms is associated with higher dental costs. Studies of the healthcare costs among smokers have received attention from researchers. Ide et al. reported that smoking could be the cause of increased dental care utilization and cost through the deterioration of oral health^11^. Based on a cohort study in Finland, current smokers were more likely to use healthcare services and visit a dentist with increased healthcare costs than never-smokers^12^. Expenditure associated with smoking includes not only direct dental costs but also indirect costs such as loss of time spent on travel and treatment with lost work time^13^. Some factors were associated with dental care utilization, such as characteristics, health beliefs, financial resources, and access to health insurance^14^.

In the Japanese medical insurance system, all Japanese residents enroll in an insurance group depending on the individual's age and occupation^15^. The healthcare system includes Employees' Health Insurance, which covers employees of private companies and their families^16^. Those who work for small and medium-sized enterprises and their families are members of the National Health Insurance Association. Under the Japanese free access healthcare system, individuals can consult multiple medical institutions, including dental clinics and hospitals^17^. All insured people and their dependents over 40–74 years can undergo specific health checkups and receive health guidance every year. From 2008, health checkups to prevent metabolic syndrome have been conducted, according to the recommendations of the Health and Medical Service Law for the Aged^18^. They provide valuable information, such as smoking status, to prevent non-communicable diseases.

Little research has been conducted on dental care utilization and cost using the Japanese administrative claims database. This study aims to evaluate the association between smoking habits and dental care utilization and cost among participants registered with the Japan Health Insurance Association, Osaka branch.

## Methods

### Data source

**Fig. 1:**
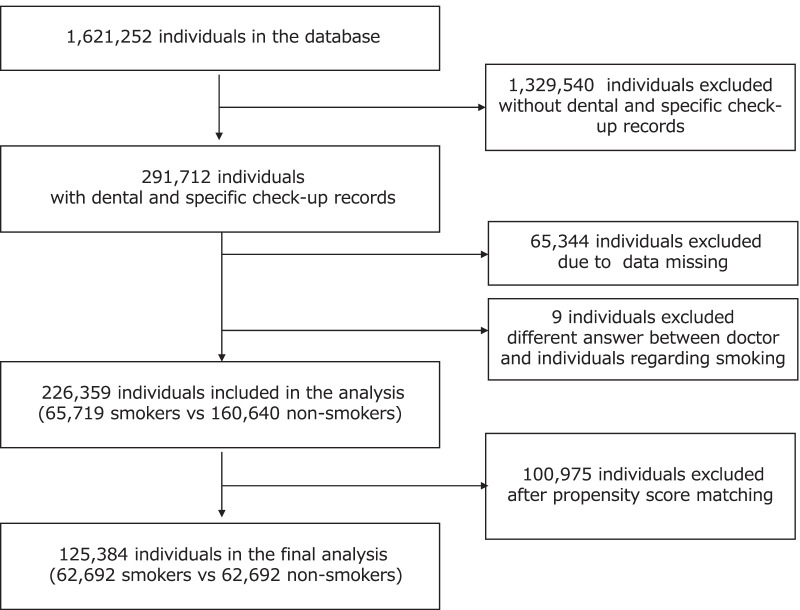
Study flow diagram

A cross-sectional study was conducted using data from the administrative claims database and specific check-up data obtained from the Japan Health Insurance Association, Osaka branch. The population in the Japan Health Insurance Association covers almost 40 million people; it is the largest medical insurer in Japan. A member firm of the Osaka Branch is a small- or medium-sized business with several small offices; approximately 80% of the offices have fewer than nine employees. The study participants were collected from April 2016 to March 2017. The participants aged older than 20 years, individuals with specific check-up records, and at least one dentist visit were included. The exclusion criteria were as follows: individuals who had missing data in the questionnaire regarding specific check-up and those whose questionnaire about smoking had answers that differed from the doctor's interview. A flow diagram of the study participants is shown in Fig. 1. Of the 1,621,252 individuals from the database of the Japan Health Insurance Association, Osaka branch, our final analytic sample consisted of 226,359 participants (from April 2016 to March 2017). Among them, 29.0% were smokers.

### Questionnaire and clinical parameters in specific medical checkup

The smoking status was determined during the medical consultation. Moreover, we also took into consideration the participant’s answer about smoking status. The questionnaire included the question, “Currently, do you smoke habitually?” (Yes/No). Currently habitual smokers were “those who have smoked a total of 100 or more cigarettes, or 6 months or longer, and have smoked in the last month.” Information of medication (hypotensive, insulin or hypoglycemic, hyperlipidemia drugs) and medical history (stroke, heart disease, chronic renal failure or artificial dialysis, anemia and metabolic syndrome) were obtained from the personal questionnaire. Further, we included other items, such as the following questions: “Have you gained over 10 kg from your weight at age 20?” (Yes/No); “Are you in the habit of exercising to sweat lightly for over 30 min a time, 2 times weekly, for over a year?” (Yes/No); “In your daily life, do you walk or do an equivalent amount of physical activity more than one hour a day?” (Yes/No); “Is your walking speed faster than the speed of those of your age and sex?” (Yes/No); “Have you gained or lost over 3 kg from your weight in a year?” (Yes/No); “Is your eating speed faster than others?” (Fast/ Normal/ Slow); “Do you skip breakfast more than 3 times a week?” (Yes/No); “Do you have any snacks or sweet beverages after dinner more than 3 times a week?” (Yes/No); “Do you have an evening meal within 2 h before bedtime more than 3 times a week?” (Yes/No); “How often do you drink alcohol?” (Everyday/ Sometimes/ Rarely); and “Do you feel refreshed after a night’s sleep?” (Yes/No). Furthermore, the check-up variables included waist circumstance (WC), body mass index (BMI), systolic blood pressure (SBP), diastolic blood pressure (DBP), total cholesterol, triglycerides, high-density lipoprotein cholesterol (HDL), low-density lipoprotein cholesterol (LDL), fasting blood glucose (FBG), HbA1c, glutamic-oxaloacetic transaminase (GOT), glutamic pyruvic transaminase (GPT), ɤ-glutamyl transpeptidase (ɤ-GTP), uric acid (UA), and estimated glomerular filtration rate (eGFR).

### Dental and medical utilization and cost

The data were linked with the claims database file using participants’ ID numbers. We also calculated dental and medical care utilization using the total annual cost of each procedure from April 2016 to March 2017. The annual number of medical visits was calculated for each participant. We counted the number of all diagnosed diseases and procedure codes of each group and calculated the prevalence between smokers and non-smokers.

### Propensity score matching and statistical analysis

We calculated propensity score, defined as the conditional probability of each participant having a smoking habit given several confounders, such as age, sex, exercise, physical activity, walking speed, eating habit (speed, breakfast, snacks, and evening meal), alcohol intake, and sleep using logistic regression (Table 1). Standardized differences were calculated to assess the balance of covariates between smokers and non-smokers. If the standardized differences were less than 10%, the covariates were considered balanced^19^. The propensity score of the smoking group and the non-smoking group were compared to create matched pairs (smokers and non-smokers as reference) within a 0.15 caliper. In this propensity score model, goodness of fit was secured (C-index was 0.699). The Student’s t-tests and the Wilcoxon–Mann–Whitney test for continuous measures. A chi-squared test was used to analyze categorical variables. A chi-squared test was used to analyze categorical variables. The 2-sided significance level was set at *P* < 0.05. All statistical analyses were performed using SPSS version 23 (IBM Corp. Armonk, NY, USA).

**Table 1:** Variables in the propensity score model

Variables
Sex
Age
Exercising
Walking/physical activity
Walking speed
Meat speed
Dinner just before going to bed
Snack after dinner
Skip the breakfast
Alcohol
Good sleep

## Results

### Demographic and clinical characteristics of participants

Table 2 shows the characteristics of smokers and non-smokers before and after propensity score matching. Compared to non-smokers, smokers were younger, and a higher proportion were men. The prevalence of anemia was higher in non-smokers (5.5% in smokers versus 10.0% in non-smokers). The results also indicate the differences in lifestyle between smokers and non-smokers. Smokers tend to skip breakfast, have dinner just before bed, and drink alcohol. Metabolic syndrome participants were 15.8% in smokers and 12.7% in non-smokers. According to propensity score matching, 62,692 participants were selected for each group. The two groups became well-balanced after propensity score matching according to standardized differences (bold letter). Clinical parameters and applicable metabolic syndrome are shown in Table 3. Smokers had a larger WC, triglyceride, ɤ-GTP and eGFR, and lower levels of total cholesterol and HDL. Standardized differences were higher than 0.1 in SBP, DBP, total cholesterol, triglyceride, HDL, and eGFR. However, the rate of metabolic syndrome was well-balanced between the two groups.

**Table 2:** Characteristics of study participants from check-up questionnaire before and after matching

	Before matching					After matching				
	Smoker		Non-smoker		S.d	Smoker		Non-smoker		S.d
Total participants	65,719		160,640			62,692		62,692		
Male (n, %)	52,933	80.5	93,326	58.1	**0.50**	49,906	79.6	49,280	78.6	**0.02**
Age (mean, SD)	49.4	9.3	50.8	9.8	0.14	49.6	9.3	49.8	9.7	0.02
*Medication (n, %)*										
Hypotensive drug	8,254	12.6	23,209	14.4	0.05	8,003	12.8	9,374	15.0	0.06
Hypoglycemic drug	2,941	4.5	6,446	4.0	0.02	2,887	4.6	2,615	4.2	0.02
Hypolipidemic drug	4,200	6.4	14,153	8.8	0.09	4,101	6.5	5,192	8.3	0.07
*Medical history (n, %)*										
Stroke	533	0.8	1,871	1.2	0.04	516	0.8	762	1.2	0.04
Heart disease	1,449	2.2	4,499	2.8	0.04	1,391	2.2	1,894	3.0	0.05
Kidney failure	170	0.3	584	0.4	0.02	164	0.3	226	0.4	0.02
Anemia	3,628	5.5	16,018	10.0	**0.17**	3,538	5.6	4,077	6.5	**0.04**
Metabolic Syndrome	10,404	15.8	20,412	12.7	0.09	9,938	15.9	9,749	15.6	0.01
*Questionnaire (n, %)*										
Body weight increase (from 20 years old)	27,975	42.6	59,974	37.3	**0.11**	26,555	42.4	27,409	43.7	**0.03**
Exercising	13,271	20.2	38,258	23.8	0.09	12,970	20.7	13,360	21.3	0.01
Walking/physical activity	23,895	36.4	60,929	37.9	0.03	22,891	36.5	23,100	36.8	0.01
Walking speed (fast)	31,648	48.2	79,509	49.5	0.03	30,321	48.4	30,507	48.7	0.01
Body weight gain and loss (in a year)	20,974	31.9	44,620	27.8	0.09	19,820	31.6	19,263	30.7	0.02
Meat speed (fast)	24,047	36.6	55,292	34.4	0.05	22,827	36.4	22,901	36.5	0.00
Dinner just before going to bed	30,471	46.4	53,773	33.5	**0.27**	28,199	45.0	27,422	43.7	**0.03**
Snack after dinner	12,308	18.7	32,984	20.5	0.05	11,818	18.9	12,075	19.3	0.01
Skip the breakfast	23,516	35.8	25,316	15.8	**0.47**	20,489	32.7	19,120	30.5	**0.05**
Alcohol (every day)	27,899	42.5	45,841	28.5	**0.30**	25,914	41.3	24,832	39.6	**0.03**
Good sleep	35,783	54.4	91,508	57.0	0.05	34,399	54.9	34,834	55.6	0.01

*S.d.* Standardizes differences; *SD* Standard deviation

**Table 3:** Clinical parameters and applicable of metabolic syndrome

	Before matching					After matching				
	Smoker		Non-smoker		S.d	Smoker		Non-smoker		S.d
Total participants	65,719		160,640			62,692		62,692		
*Clinical parameter (mean, SD)*										
WC (cm)	83.4	10.1	82.2	10.1	0.13	83.4	10.2	83.7	9.9	0.03
BMI (kg/m2)	23.3	3.7	23.1	3.7	0.07	23.3	3.7	23.5	3.6	0.06
SBP (mmHg)	121.7	17.7	121.6	18.0	0.01	121.6	17.7	123.4	17.6	0.10
DBP (mmHg)	75.4	12.5	75.1	12.5	0.02	75.3	12.5	76.8	12.4	0.12
Total cholesterol (mg/dl)	202.6	34.7	206.7	34.3	0.12	202.7	34.7	206.6	34.4	0.11
Triglyceride (mg/dl)	134.7	121.6	104.5	88.9	0.28	134.3	121.6	115.1	103.2	0.17
HDL (mg/dl)	57.6	24.3	64.8	16.9	0.34	57.7	24.6	62.4	16.4	0.23
LDL (mg/dl)	122.4	32.8	123.0	31.1	0.02	122.5	32.8	123.7	31.3	0.04
FBG (mg/dl)	98.1	23.0	96.6	18.7	0.07	98.1	23.0	98.0	19.6	0.01
HbA1c (%)	5.6	0.8	5.6	0.7	0.06	5.6	0.8	5.6	0.7	0.05
GOT (U/L)	23.2	13.0	23.2	11.6	0.01	23.2	12.9	24.1	11.4	0.08
GPT (U/L)	24.7	18.7	23.1	18.2	0.09	24.6	18.6	25.5	18.7	0.05
γ-GTP (U/L)	49.4	65.7	38.4	48.1	0.19	48.8	64.1	45.8	56.8	0.05
UA (mg/dl)	5.7	1.4	5.4	1.4	0.22	5.7	1.4	5.8	1.4	0.07
CRE (mg/dl)	0.8	0.4	0.8	0.3	0.04	0.8	0.5	0.8	0.3	0.08
eGFR (ml/min/1.73m2)	81.0	14.3	77.4	14.2	0.25	80.8	14.3	77.6	13.9	0.23

*S.d.* Standardizes differences; *SD* Standard deviation; *WC* Waist circumstance; *BMI* body mass index; *SBP* Systolic blood pressure; *DBP* Diastolic blood pressure; *FBG* Fasting blood sugar; *GOT* Glutamic oxaloacetic transaminase; *GPT* Glutamic pyruvic transaminase; *γ-GTP* γ-glutamyltranspeptidase; *UA* Uric acid; *eGFR* estimated glomerular filtration rate

### Dental and medical care utilization and cost between smokers and non-smokers

As shown in Table 4, after adjusting for potential confounding factors with propensity score matching, the mean annual dental cost was 54,520 JPY (Standard deviation (SD): 54,774) among smokers, significantly higher than non-smokers (47,944 JPY; SD: 50,951) (*P* < 0.001). Of the 125,384 participants, 19.0% of the smokers and 13.6% of the non-smokers did not visit medical institutions. Non-smokers tend to see medical doctors with higher medical cost.

**Table 4:** Dental and medical utilization and cost between smokers and non-smokers

	Smoker		Non-smoker		*P*
	62,692		62,692		
*Annual dental cost (JPY)*					
Mean, SD	54,520	54,774	47,944	50,951	< 0.001
Median, 1Q,3Q	38,750	19,210, 71,000	34,740	18,090, 61,450	< 0.001
*Number of dental institutions*					
Mean, SD	3.7	2.7	3.7	2.6	< 0.001
Median, 1Q,3Q	3	2, 5	3	2, 5	< 0.001
*Annual medical cost (JPY)*					
Mean, SD	99,916	372,926	113,479	382,906	< 0.001
Median, 1Q,3Q	27,010	5,580, 80,720	37,020	9470, 96,550	< 0.001
*Number of medical institutions*					
Mean, SD	5.6	6.1	6.8	6.9	< 0.001
Median, 1Q,3Q	3	1, 9	5	2, 10	< 0.001

Student’s t-tests Median (Interquartile range (IQR)), Wilcoxon-Mann–Whitney test

*SD* Standard deviation; *1Q* 1st quartile; *3Q* 3rd quartile;

### Dental diagnosis and dental procedure between smokers and non-smokers

Pulpitis, missing teeth, and apical periodontitis were more frequently diagnosed in smokers, while inlay detachment, dental decay, and hypersensitivity were more common in non-smokers (Table 5). As for dental procedures, smokers needed more frequent dental recalls and medication-assisted treatment to control acute inflammation (as shown in Table 6). However, the first consultation that included the Basic Periodontal Examination and scaling procedures were more frequently coded in non-smokers.

**Table 5:** Dental diagnosis between smokers and non-smokers

	Smoker		Non-smoker		*P*-value
	62,692		62,692		
*Dental diagnosis (n, %)*					
Periodontitis	47,182	75.3	47,881	76.4	< 0.001
Caries	28,697	45.8	29,764	47.5	< 0.001
Pulpitis	8,999	14.4	6,132	9.8	< 0.001
Apical periodontitis	8,069	12.9	5,721	9.1	< 0.001
Missing tooth	7,433	11.9	4,605	7.3	< 0.001
Caries treated teeth	5,802	9.3	4,741	7.6	< 0.001
Hypersensitivity	5,132	8.2	5,849	9.3	< 0.001
Dentin caries	5,153	8.2	5,541	8.8	< 0.001
Acute chronic marginal periodontitis	4,580	7.3	3,464	5.5	< 0.001
Incompatible denture	3,865	6.2	2,783	4.4	< 0.001
Acute purulent periodontitis	3,826	6.1	2,844	4.5	< 0.001
Acute apical periodontitis	3,784	6.0	2,803	4.5	< 0.001
Missing tooth, bridge	3,435	5.5	2,304	3.7	< 0.001
Inlay detachment	2,266	3.6	3,424	5.5	< 0.001
Temporomandibular disorders	1,502	2.4	1,762	2.8	< 0.001
All metal crown desorption	1,302	2.1	1,367	2.2	= 0.203
Bruxism	972	1.6	1,326	2.1	< 0.001
Enamel early caries	577	0.9	711	1.1	< 0.001
Chronic marginal periodontitis	444	0.7	481	0.8	= 0.222
Aphthous stomatitis	307	0.5	429	0.7	< 0.001

Chi-squared test

**Table 6:** Dental procedure between smokers and non-smokers

	Smoker		Non-smoker		*P*-value
	62,692		62,692		
*Dental procedure (n, %)*					
Initial consultation	51,230	81.7	52,138	83.2	< 0.001
Reexamination	50,082	79.9	47,968	76.5	< 0.001
Professional mechanical tooth cleaning	43,150	68.8	45,150	72.0	< 0.001
Scaling	42,810	68.3	45,458	72.5	< 0.001
Basic periodontal examination	40,986	65.4	44,338	70.7	< 0.001
Dental hygiene practical guidance	38,260	61.0	40,559	64.7	< 0.001
Prescription	23,138	36.9	16,907	27.0	< 0.001
Dispensing	22,417	35.8	15,987	25.5	< 0.001
Drug information provision	22,197	35.4	16,234	25.9	< 0.001
Dental X-ray	19,166	30.6	16,678	26.6	< 0.001
Photo diagnosis	19,148	30.5	16,656	26.6	< 0.001
Electrical root canal length measurement test	15,116	24.1	11,417	18.2	< 0.001
Crown preparation	14,194	22.6	11,219	17.9	< 0.001
Inlay restoration	9,821	15.7	11,061	17.6	< 0.001
Molar tooth extraction	9,109	14.5	6,050	9.7	< 0.001
Inlay preparation	8,291	13.2	9,450	15.1	< 0.001
Intracanal medication	7,549	12.0	5,924	9.4	< 0.001
Periodontal inspection	7,037	11.2	7,915	12.6	< 0.001
Replacement	4,521	7.2	5,680	9.1	< 0.001
Regional dental care support hospital reexamination	912	1.5	1,086	1.7	< 0.001

Chi-squared test

## Discussion

In the present study, we show a positive association between smoking habit and high dental cost. Smokers were more likely to incur dental care costs than non-smokers among the users of dental and medical check-up services. Warnakulasuriya et al. have shown that oral health risk such as oral cancer, periodontal disease, tooth loss, implant failure, and dental caries have a strong association with tobacco smoking^20^. The cost of smoking exceeds the cost of periodontal therapy^21^. However, we could not obtain information about daily tooth cleaning habits, such as the frequency of using dental floss or other types of teeth cleaning tools. The result of the present study is consistent with that of a report that current smokers were less likely to consult a dentist in the past year than those who never smoked^8^. There was no significant association between children’s exposure to secondhand smoke and dental expenditure using the Medicaid database^22^. It is also necessary to consider the impact of secondhand smoke on medical and dental costs.

In our results, smokers were more susceptible to progressed dental caries, missing teeth, and uncontrolled acute inflammation. Therefore, the rate of recalls and need for medications to control acute dental inflammation was higher in smokers. Non-smokers were more likely to receive the first consultation, including the Basic Periodontal Examination, and the scaling procedure as part of the routine dental examination. Some reports showed that, compared with never smokers, current smokers were less likely to exercise daily preventive care^8^.

Based on the Act on Ensuring Medical Care for the Elderly, the Specified Health Checkup was started in 2008. In 2016, the targeted population was 53.6 million individuals with basic insurance, aged 40 to 74 years old; the total average rate of those who received this checkup was 51.4%. Although the consultation rate increased over time, the financial situation of the insurer remained variable. More than 75% of insured individuals underwent specific checkups in the health insurance society, but less than 50% of insurers received checkups in the National Insurance Association^23^. Individuals who have not undergone routine medical checkups are included in the list.

The data of participants in this study were derived from the administrative claims database and specific checkups. The advantage of a real-world database, such as the medical claims database used in our study, is that it can provide diagnosis and treatment information even if a participant switches to another medical institution^24^. Moreover, we have used a propensity score matching method to adjust for potential confounding variables, such as lifestyle between the two groups.

There are some limitations to this study. We could only consider the medical and dental service under health insurance due to the characteristics of the administrative claims database. Individuals who did not visit the dental clinic were not analyzed. Therefore, medical expenses may have been overestimated. Free dental treatments, such as whitening, orthodontic treatment, implants, and aesthetic dentistry, were not included in this analysis. According to a survey conducted by the Ministry of Health, Labour and Welfare, the implementation rate of specific checkups was 47.4% in all Japan Health Insurance Associations. Hence, the utilization of medical and dental services may have been overestimated considering the total population. The information regarding pharmacy claims was not included in this database. Thus, the cost of healthcare excluded medication costs. We could not distinguish between former smokers and non-smokers without data on the smoking period. Jeong reported that electronic cigarette vaping and conventional cigarette use had a significant association with periodontal disease rates^25^. However, we were unable to identify the type of cigarettes used in this study. Socioeconomic status is a well-known risk indicator for dental disease^26^. The study population comprised insured individuals who were enrolled in the Japan Health Insurance Association, Osaka branch. However, we could not evaluate the economic situation in each household. Adults 75 years or older with high medical and dental costs could not be included in this study, as they usually transfer to the medical care system for the elderly.

According to National Health and Nutrition Survey, smoking habits are declining among both men and women since 2016 (30.2% in men and 7.2% in women). Although smokers are at a higher risk of oral pain, smoking cessation significantly decreases the risk^27^. The adverse effects of smoking on oral health are often reduced by smoking cessation. As there exists a strong dose-dependent association between smoking and tooth loss, dentists play a crucial role in tobacco control^2^.

In summary, this study suggests that smokers have higher dental costs, most often due to acute illness. Our findings support the call for public policies to promote non-smoking activities. Promoting regular dental visits is one strategy to help people prevent and treat oral disease in early stages.

## Conclusion

The annual dental cost was significantly higher among smokers than non-smokers. The disease codes in smokers consisted of progressive dental caries, missing teeth, and uncontrolled acute inflammation. It is suggested that smokers tend to visit the dentist after their symptoms become severe.

## Data Availability

The data that support the findings of this study are available from the Japan Health Insurance Association. However, restrictions apply to the availability of these data, which were used under license for the current study, and hence, are not publicly available. Data are, however, available from the authors upon reasonable request and with permission of the Japan Health Insurance Association.

## References

[1] National Health and Nutrition Survey in 2018. Ministry of Health, Labour, and Welfare, Japan. https://www.mhlw.go.jp/content/10904750/000351576. Accessed April 26, 2022.

[2] World Health Organization. WHO Report on the Global Tobacco Epidemic, 2017 external icon. Geneva: World Health Organization, 2017. https://www.who.int/publications/i/item/9789241512824. Accessed April 26, 2022.

[3] Dietrich T, Walter C, Oluwagbemigum K, Bergmann M, Pischon T, Pischon N, Boeing H (2015). Smoking, smoking cessation, and risk of tooth loss: The EPIC-Potsdam Study. J Dent Res.

[4] American Academy of Periodontology (AAP). Position paper: tobacco use and the periodontal patient. Research, science and therapy committee of the American Academy of Periodontology. *J Periodontol*. 1999;70(11):1419–1427. 10.1902/jop.1999.70.11.1419 10588507

[5] Adeoye J, Hui L, Tan JY, Koohi-Moghadam M, Choi SW, Thomson P (2021). Prognostic value of non-smoking, non-alcohol drinking status in oral cavity cancer. Clin Oral Investig.

[6] Tanaka S, Shinzawa M, Tokumasu H, Seto K, Tanaka S, Kawakami K (2015). Secondhand smoke and incidence of dental caries in deciduous teeth among children in Japan: population based retrospective cohort study. BMJ.

[7] Drilea SK, Reid BC, Li CH, Hyman JJ, Manski RJ (2005). Dental visits among smoking and nonsmoking US adults in 2000. Am J Health Behav.

[8] Millar WJ, Locker D (2007). Smoking and oral health status. J Can Dent Assoc..

[9] Csikar J, Kang J, Wyborn C, Dyer TA, Marshman Z, Godson J (2016). The self-reported oral health status and dental attendance of smokers and non-smokers in England. PLoS ONE.

[10] Mucci LA, Brooks DR (2001). Lower use of dental services among long term cigarette smokers. J Epidemiol Community Health.

[11] Ide R, Hoshuyama T, Wilson D, Takahashi K, Higashi T (2009). The effects of smoking on dental care utilization and its costs in Japan. J Dent Res.

[12] Keto J, Ventola H, Jokelainen J, Timonen M, Linden K, Ylisaukko-Oja T, Keinanen-Kiukaanniemi S, Auvinen J (2017). Primary health care utilisation and its costs among middle-aged smokers. Eur J Health Econ.

[13] Park YD, Kang JO, Kim SJ, Kwon HJ, Hwang JH, Hwang KS (2012). Estimation of the costs of smoking-related oral disease: a representative South Korean study. Int Dent J.

[14] Blasi PR, Krakauer C, Anderson ML, Nelson J, Bush T, Catz SL, McClure JB (2018). Factors associated with future dental care utilization among low-income smokers overdue for dental visits. BMC Oral Health.

[15] Okamura S, Kobayashi R, Sakamaki T (2005). Case-mix payment in Japanese medical care. Health Policy.

[16] Yoshida M, Takada T, Hirata K, Mayumi T, Shikata S, Shirai K, Kimura Y, Wada K, Amano H, Arata S, Hirota M, Takeda K, Gabata T, Hirota M, Yokoe M, Kiriyama S, Sekimoto M (2010). Health insurance and payment systems for severe acute Pancreatitis. J Hepatobiliary Pancreat Sci..

[17] Suzuki T, Iwagami M, Hamada S, Matsuda T, Tamiya N (2020). Number of consulting medical institutions and risk of polypharmacy in community-dwelling older people under a healthcare system with free access: a cross-sectional study in Japan. BMC Health Serv Res.

[18] Ministry of Health, Labour, and Welfare, Japan. Standard program of health checkup and health guidance. https://www.mhlw.go.jp/bunya/shakaihosho/iryouseido01/info03a.html. Accessed April 26, 2022.

[19] Cohen J (1988). Statistical power analysis for the behavioural sciences.

[20] Warnakulasuriya S, Dietrich T, Bornstein MM, Casals Peidro E, Preshaw PM, Walter C, Wennstrom JL, Bergstrom J (2010). Oral health risks of tobacco use and effects of cessation. Int Dent J.

[21] Fardal O, Grytten J, Martin J, Ellingsen S, Fardal P, Heasman P, Linden GJ (2018). Adding smoking to the Fardal model of cost-effectiveness for the lifetime treatment of periodontal diseases. J Periodontal.

[22] Levy DE, Rigotti NA, Winickoff JP (2011). Medicaid expenditures for children living with smokers. BMC Health Serv Res.

[23] Ministry of Health, Labour, and Welfare, Japan. The situation of receiving health checkup and health guidance. https://www.mhlw.go.jp/content/12400000/000340076.pdf. Accessed December 19, 2019.

[24] Tanaka S, Seto K, Kawakami K (2015). Pharmacoepidemiology in Japan: medical databases and research achievements. J Pharm Health Care Sci.

[25] Jeong W, Choi DW, Kim YK, Lee HJ, Lee SA, Park EC, Jang SI (2019). Associations of electronic and conventional cigarette use with periodontal disease in South Korean adults. J Periodontal.

[26] Lu J, Zaimi I, Barber JR, Joshu CE, Prizment AE, Beck JD, Platz EA, Michaud DS (2019). SES and correlated factors do not explain the association between periodontal disease, edentulism, and cancer risk. Ann Epidemiol.

[27] Riley JL, Tomar SL, Gilbert GH (2004). Smoking and smokeless tobacco: increased risk for oral pain. J Pain.

